# Integrated Analysis Identifies an Anoikis‐Related Gene Signature for Predicting Prognosis in Patients With Triple‐Negative Breast Cancer

**DOI:** 10.1049/syb2.70076

**Published:** 2026-06-08

**Authors:** Bing Xue, Yue Bai, Wu‐Di Yang, Shi‐Zhen Li, Rui Qin, Hua Zhang, Hong‐Fei Wu, Jun‐Jian Li, Sen Miao

**Affiliations:** ^1^ Department of Pathology Affiliated Hospital of Jining Medical University Jining China; ^2^ School of Clinical Medicine Jining Medical University Jining China; ^3^ Department of Pathology Shandong Public Health Clinical Center Jinan China; ^4^ Department of Pathology Yantai Yuhuangding Hospital Yantai China; ^5^ Department of Vascular Surgery Hainan General Hospital (Hainan Affiliated Hospital of Hainan Medical University) Haikou China; ^6^ State Key Laboratory of Systems Medicine for Cancer Department of Oncology Shanghai General Hospital Shanghai Jiao Tong University School of Medicine Shanghai China

**Keywords:** bioinformatics, genomics, patient treatment, tumours

## Abstract

Triple‐negative breast cancer (TNBC) is an aggressive breast cancer subtype with limited therapeutic options and poor prognosis. Anoikis resistance contributes to tumour dissemination, metastasis and therapeutic resistance. However, anoikis‐related molecular signatures integrating single‐cell and bulk transcriptomic data for prognostic prediction in TNBC remain insufficiently characterised. Bulk RNA‐seq data from TCGA–TNBC and GSE58812, together with single‐cell transcriptomic data, were analysed. Anoikis‐related genes were identified by WGCNA and single‐cell module scoring. A prognostic signature was constructed using Cox and LASSO regression. Immune features and immunotherapy‐related indicators were evaluated, and STC2 was validated by tissue microarray, immunohistochemistry and in vitro anoikis assays. An anoikis‐related gene signature (*ASS1*, *COL18A1*, *STC2*, *DHX36*, *S100B* and *TGFBI*) was established. High‐risk patients exhibited poor overall survival, increased cancer‐associated fibroblast infiltration and reduced T‐cell infiltration. Additionally, high anoikis risk scores were associated with increased mutations frequencies of *PTEN*, *PIK3CA* and *ZEB2*, along with lower tumour mutation burden, microsatellite instability and PD‐L1 levels. IHC staining confirmed that patients with low STC2 expression had poor prognosis. Furthermore, STC2 knockdown reduced anoikis‐related apoptotic rates in an MDA‐MB‐231‐based anoikis‐mimic model in vitro. This study established an anoikis‐related gene signature that may improve prognostic stratification and reflect immunotherapy‐related features in TNBC.

## Graphical abstract

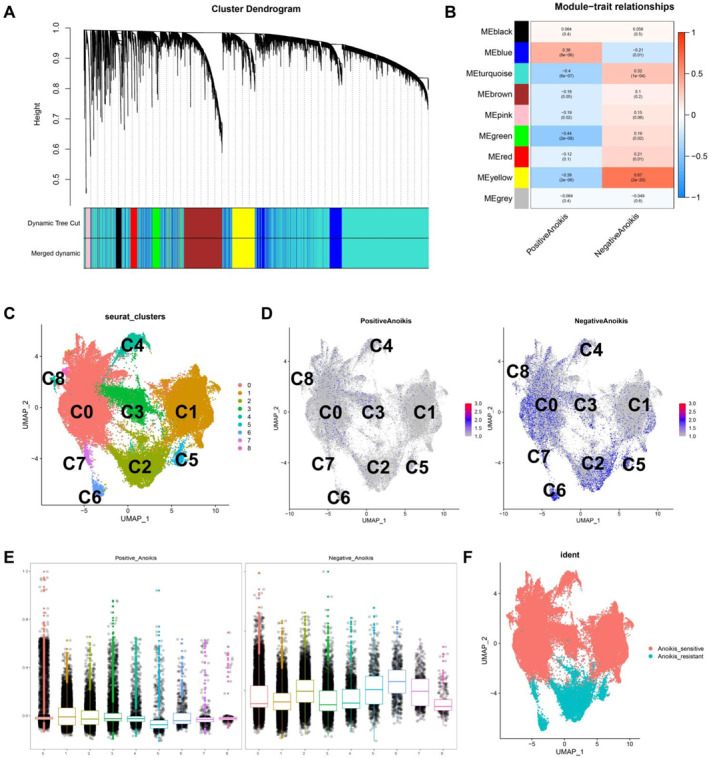

## 1 Introduction

Triple‐negative breast cancer (TNBC) is an aggressive breast cancer subtype linked to poorer clinical outcomes compared to other subtypes, defined by the absence of oestrogen receptor (ER), progesterone receptor and human epidermal growth factor receptor 2 (HER2) expression. Consequently, standard endocrine and anti‐HER2 therapies are ineffective for treating TNBC [1, 2, 3, 4, 5, 6]. Chemotherapy remains the standard postoperative treatment for TNBC, and its combination with immunotherapy has shown promise in improving clinical outcomes [7, 8, 9, 10]. However, many women globally remain resistant to chemotherapy and continue to succumb to TNBC each year. This necessitates the urgent identification of molecular indicators capable of predicting therapeutic responses and patient outcomes.

Anoikis describes a specific type of programed cell death occurring upon cellular detachment from the extracellular matrix (ECM) [11, 12]. This process serves as a crucial mechanism in tissue equilibrium maintenance by eliminating displaced cells [11]. In certain cancers, however, detached tumour cells can evade anoikis, enabling their survival in suspension and contributing to cancer invasion and metastasis [12]. Multiple studies have highlighted the association between anoikis resistance and metastasis as well as chemoresistance. Factors, such as BMP4, PRKCQ, IL17A, TGFB1 and DBC1, have been linked to anoikis resistance in TNBC [13, 14, 15, 16, 17, 18, 19, 20]. Despite this, the molecular signature associated with anoikis for predicting treatment response and prognosis in TNBC remains insufficiently defined.

Single‐cell RNA sequencing (scRNA‐seq) has evolved into an essential methodology for examining transcriptional patterns across distinct cellular populations [21]. Recent developments in sequencing technologies enable detailed examination of gene expression at individual cell resolution, revealing tumour complexity and cell‐type variation. Multiple research initiatives have subsequently combined scRNA‐seq with conventional RNA‐seq methodologies to discover novel cancer biomarkers [22]. This investigation seeks to establish a predictive framework for patients with TNBC through the integration of scRNA‐seq and bulk RNA‐seq information, confirming its stratification effectiveness across two independent patient groups. These findings could reveal potential anoikis‐related prognostic indicators and treatment targets specific to TNBC.

This investigation developed an anoikis‐related signature to forecast TNBC patient outcomes through the integration of single‐cell and bulk RNA‐seq analyses. These results may facilitate the identification of patients with TNBC who could derive optimal benefit from chemotherapeutic or immunotherapeutic interventions.

## 2 Materials and Methods

### 2.1 Data Acquisition

The UCSC Xena data portal (https://xenabrowser.net) provided transcriptomic information from The Cancer Genome Atlas–BRCA (TCGA–BRCA) cohort [23]. The GEO database (https://www.ncbi.nlm.nih.gov/geo/) supplied transcriptomic data from GSE58812 (107 samples) [24] and additional cohorts. Single‐cell information was procured through http://biokey.lambrechtslab.org [25]. For 146 TNBC samples in the TCGA–TNBC dataset, anoikis‐related scores (positive and negative) were computed using single‐sample gene set enrichment analysis (ssGSEA) through the ‘GSVA’ R package based on genes that positively and negatively regulate anoikis. Gene Ontology (http://amigo.geneontology.org/amigo) provided gene lists for positive and negative anoikis regulation under accession numbers GO:2000210 and GO:2000811 [26, 27, 28]. The Cancer Immunome Atlas (https://tcia.at/home) supplied immunophenoscores (IPS) for immunotherapy response prediction [29, 30].

### 2.2 Weighted Correlation Network Analysis (WGCNA)

Weighted gene co‐expression network analysis (WGCNA) was conducted using TCGA–TNBC transcriptomic data with R software version 4.1.0. The main parameters were set as follows: soft‐thresholding power *β* = 3, minimum module size = 200 and module merge cut height = 0.25. Pearson correlation analysis was used to identify anoikis‐related modules (|coefficient| > 0.3, *p* < 0.05). According to the gene co‐expression network clustering results of WGCNA, we further screened and defined two distinct gene sets, including the positive anoikis gene set (|coefficient|> 0.3, *p* < 0.05) and the negative anoikis gene set (coefficient < −0.3, *p* < 0.05), which were used for subsequent anoikis resistance signature evaluation at the single‐cell level.

### 2.3 Single‐Cell Sequencing Data Analysis

Cancer cells from patients with TNBC in Bassez cohort 1 were analysed using the Seurat package in R software version 4.1.0 [31]. Batch effects were corrected using the harmony package [32]. Strict quality control was performed to exclude low‐quality cells, empty droplets and potential doublets. Cells were retained only if they met the following criteria: mitochondrial gene expression fraction ≤ 15%, number of detected genes ranging from 200 to 8000 and total UMI counts ranging from 500 to 80,000. Genes expressed in fewer than three cells were excluded. After quality control, gene expression data were normalised using the LogNormalize method with a scaling factor of 10,000. Highly variable genes were identified using the variance‐stabilising transformation method, and principal component analysis was performed based on the top 2000 highly variable genes. The top 20 principal components were used for dimensionality reduction and clustering. UMAP was used for visualisation, and graph‐based clustering was performed using Seurat. Anoikis‐related scores for individual cancer cells were calculated using the AddModuleScore function based on the positive and negative anoikis‐related gene sets. Clusters with significantly higher average scores for the positive anoikis‐related gene set than the median level across all clusters were defined as positive anoikis clusters, whereas clusters with significantly higher average scores for the negative anoikis‐related gene set were defined as negative anoikis clusters (Wilcoxon rank‐sum test, *p* < 0.05). Clusters enriched for the negative anoikis‐related signature without significant enrichment of the positive anoikis‐related signature were classified as anoikis‐resistant subtypes, whereas the remaining clusters were classified as anoikis‐sensitive subtypes. Differential markers between anoikis‐resistant and anoikis‐sensitive subtypes were then identified using the FindMarkers function with an FDR < 0.05.

### 2.4 Development and Validation of an Anoikis‐Related Genes

The TCGA–TNBC cohort mRNA expression information facilitated the development of an anoikis‐related signature to forecast overall survival (OS) among patients with TNBC. Anoikis‐related genes were defined as those present in both the anoikis‐related modules (identified through WGCNA) and the anoikis‐related marker list (from single‐cell analysis). The identification of prognostic signatures involved sequential implementation of univariate Cox regression, lasso regression and stepwise multivariate Cox regression analyses. Evaluation of the signature's prognostic significance utilised receiver operating characteristic (ROC) curve analysis and survival assessment incorporating data from both TCGA and GSE58812 cohorts. The calculation of anoikis risk scores for individual patients with TNBC proceeded as follows: Anoikis risk score = 0.607 × *ASS1* + 0.659 × *COL18A1* − 0.771 × *DHX36* − 0.250 × *S100B* − 0.605 × *STC2* + 0.375 × *TGFBI*, where each gene symbol represents the normalised expression value of the corresponding gene. The optimal cutoff for the anoikis risk score was determined using the maxstat package, and patients were stratified into high‐risk and low‐risk groups based on maximal survival separation. TME characteristics between the two groups in the TCGA–TNBC cohort were evaluated using ESTIMATE [33], Xcell [34], MCPCounter [34] and EPIC [35] implemented via the ‘IOBR’ package [36]. TME assessment involved calculating ssGSEA scores for individual immune cell populations across samples. HALLMARK gene sets, obtained from the MSigDB database [37, 38], underwent ssGSEA score computation for each TCGA–TNBC cohort sample. The analysis identified differentially expressed genes (DEGs) between risk groups using criteria of fold change (FC) ≥ 1.5 or ≤ 0.5 and FDR < 0.05. Kyoto Encyclopaedia of Genes and Genomes (KEGG) enrichment analysis incorporated these DEGs. The analytical procedures utilised Sangerbox (http://www.sangerbox.com/tool) with current R software versions [39].

### 2.5 Public Online Database Analysis

TNMplot (https://tnmplot.com/analysis/) was utilised to examine *STC2* expression in normal, tumour and metastatic breast tissues using gene chip data [40].

### 2.6 Tissue Microarray (TMA) and Immunohistochemistry (IHC)

Seventy nine breast tumour samples and 68 adjacent normal tissue specimens were obtained from the Affiliated Hospital of Jining Medical University. All specimens underwent haematoxylin and eosin (HE) staining, followed by selection of representative regions for tissue microarray (TMA) construction. The prepared TMA slides underwent overnight drying at 37°C prior to staining procedures. TMA sections received overnight incubation at 4°C with STC2 antibody (1:300, Abcam, ab63057), utilising the Ventana Ultraview Universal DAB Detection Kit (#760‐500) for visualisation. STC2 expression quantification employed an IHC scoring system derived from multiplying staining intensity by positive tumour cell percentage. The intensity classification comprised four categories: 0 (negative), 1 (weak), 2 (moderate) and 3 (strong). Positive tumour cell percentages were divided into five groups: 0 (≤ 5%), 1 (6%–25%), 2 (26%–50%), 3 (51%–75%) and and 4 (> 76%). An IHC score ≥ 6 was defined as high STC2 expression. Two independent experienced pathologists evaluated the STC2 expression (Table S1).

### 2.7 Cell Culture

The TNBC cell line MDA‐MB‐231 and the HEK293T cell line were procured from ATCC. Cells were cultured in Dulbecco's Modified Eagle Medium (DMEM; Gibco, Shanghai, China) comprising 10% foetal bovine serum (Gibco, South America) and 1% penicillin–streptomycin (Gibco, Shanghai, China) within an incubator maintaining 37°C and 5% CO_2_.

### 2.8 Cell Transfection by Lentivirus

Small hairpin RNA (shRNA) plasmids were purchased from Tsingke Biotech (Beijing, China). The PLKO.1‐shRNA, psPAX and PMD2.0g vectors were transfected into HEK293T cells using Lipofectamine 2000 (Thermo Fisher, Shanghai, China). Lentivirus supernatant was collected and filtered 48 h after medium refreshment. The lentivirus was used to transduce MDA‐MB‐231 cells, which were selected with puromycin (2 μg/mL) for at least 2 weeks. The target sequences for STC2 and negative control shRNAs (shNC) were as follows: (shNC: CGTGCCCTGGCCCACCCTCG; shSTC2#1: GAAGACGAACAGTCTGAGTAT; shSTC2#2: CCAGGGCAAGTCATTCATCAA).

### 2.9 Construction of Anoikis Model

To generate the anoikis model, 1 × 10^6^ cells were placed in ultra‐low attachment 6‐well plates (Corning, NY, USA) and incubated for 24 h. Suspended cells were collected, washed with PBS and stained with Annexin V and propidium iodide for 15 min at 4°C. The anoikis rate was quantified using a Beckman Coulter flow cytometer (USA) in the FITC and PE channels.

### 2.10 RT‐qPCR

Total RNA isolation from MDA‐MB‐231 cells was accomplished utilising TRIzol reagent (Invitrogen, Grand Island, NY, USA). The reverse transcription process employed HiScript II Q Select RT SuperMix for qPCR (Vazyme, Nanjing, China). Quantitative PCR analysis was conducted by utilising SYBR PCR Master Mix (Vazyme) on the Applied Biosystems ABI 7500 platform (Applied Biosystems, Waltham, MA, USA). GAPDH served as the housekeeping gene for normalisation. Expression changes were calculated through the 2^−ΔΔCt^ method. All experiments were performed in three independent replicates. The utilised primer sequences include: (*GAPDH*‐qForward: AGGTCGGAGTCAACGGATTT; *GAPDH*‐qReverse: ATCTCGCTCCTGGAAGATGG; *STC2*‐qForward: TCTTGTGAGATTCGGGGCTT; *STC2*‐qReverse: ACAGGTCGTGCTTGAGGTAG).

### 2.11 Western Blot Assay

Proteins were extracted using RIPA buffer, and protein concentrations were determined using a BCA protein assay kit (23227, Thermo Fisher, Shanghai, China). Protein samples (40 μg) were separated on SDS‐PAGE gels and transferred to nitrocellulose membranes (HATF00010, Sigma, Germany). The membranes were blocked with 5% nonfat milk and incubated at 4°C with primary antibodies against STC2 (1:2000, Abcam, Shanghai, China) and GAPDH (1:10000, Proteintech, Wuhan, China). Following TBST washing steps, HRP‐conjugated secondary antibody application was performed. ECL substrate was used for exposure, and signals were detected using the Tianneng automatic chemiluminescence imaging system (Tanon, Shanghai, China).

### 2.12 Statistical Analysis

Statistical comparisons between paired groups utilised a two‐tailed *t*‐test analysis. Patient survival evaluation employed log‐rank statistical testing. Statistical significance was established at *p* < 0.05.

### 2.13 Data Availability

The datasets used and/or analysed during the current study are available from the corresponding author upon reasonable request.

## 3 Results

### 3.1 Identification of Anoikis‐Related Genes in TNBC by Integrating Single‐Cell and Bulk Transcriptomic Data

**FIGURE 1:**
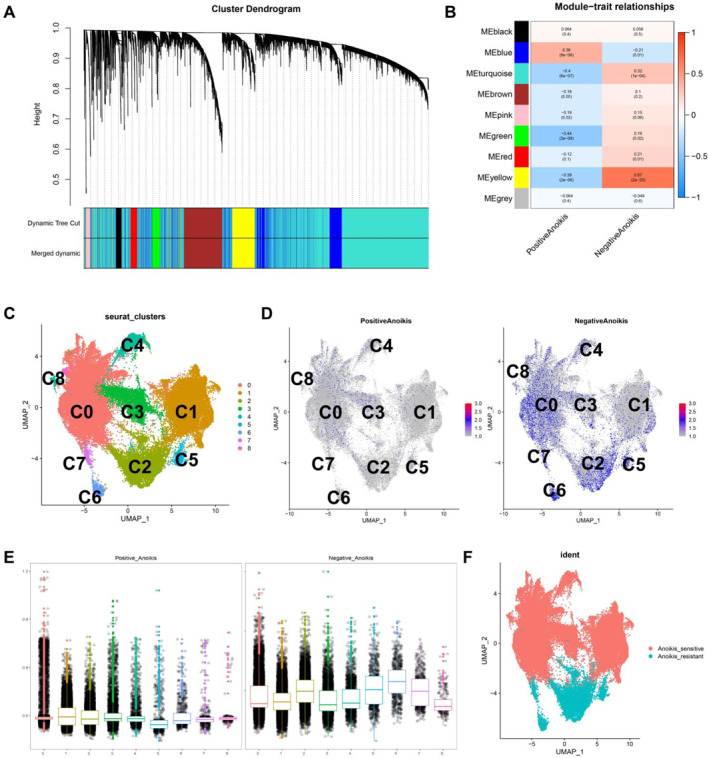
Discovery of anoikis‐related genes. (A) Dendrogram of co‐expressed clusters with 9 modules identified. (B) Heatmap showing the correlation between modules and anoikis‐related ssGSEA scores in the TCGA–TNBC cohort. (C) UMAP plot of cancer cells from Bassez cohort 1 with 9 clusters identified. (D) UMAP plot showing anoikis‐related scores in cancer cells from Bassez cohort 1. (E) Box plot of anoikis‐related scores across the 9 clusters of cancer cells in Bassez cohort 1. (F) UMAP plot of cancer cells from Bassez cohort 1, highlighting the anoikis‐resistant and anoikis‐sensitive subtypes. For TCGA–TNBC bulk transcriptomic analysis, *n* = 146 patients.

WGCNA was conducted using mRNA expression data from the TCGA–TNBC cohort. Through hierarchical clustering and dynamic tree cut approaches, the analysis revealed 9 distinct modules (Figure 1A). The yellow and turquoise modules were designated as anoikis‐related modules as both demonstrated statistically significant associations with the positive anoikis and negative anoikis scores in TNBC (Figure 1B). Concurrently, single‐cell data of cancer cells from the Bassez cohort 1 were analysed using the Seurat package, leading to the detection of 9 distinct cancer cell clusters (Figure 1C). The results showed that clusters 2, 5, 6 and 7 had significantly higher average scores for the negative anoikis gene set than the median level across all clusters (*p* < 0.05) and were defined as negative anoikis clusters. No clusters showed significantly higher scores for the positive anoikis gene set (Figure 1D). The positive and negative anoikis scores were calculated at the single‐cell level (Figure 1E). Therefore, clusters 2, 5, 6 and 7 were classified as the anoikis‐resistant subtype, and clusters 0, 1, 3, 4 and 8 were classified as the anoikis‐sensitive subtype (Figure 1F).

### 3.2 Development and Validation of an Anoikis‐Related Gene Signature for Survival Prediction in Patients With TNBC

**FIGURE 2:**
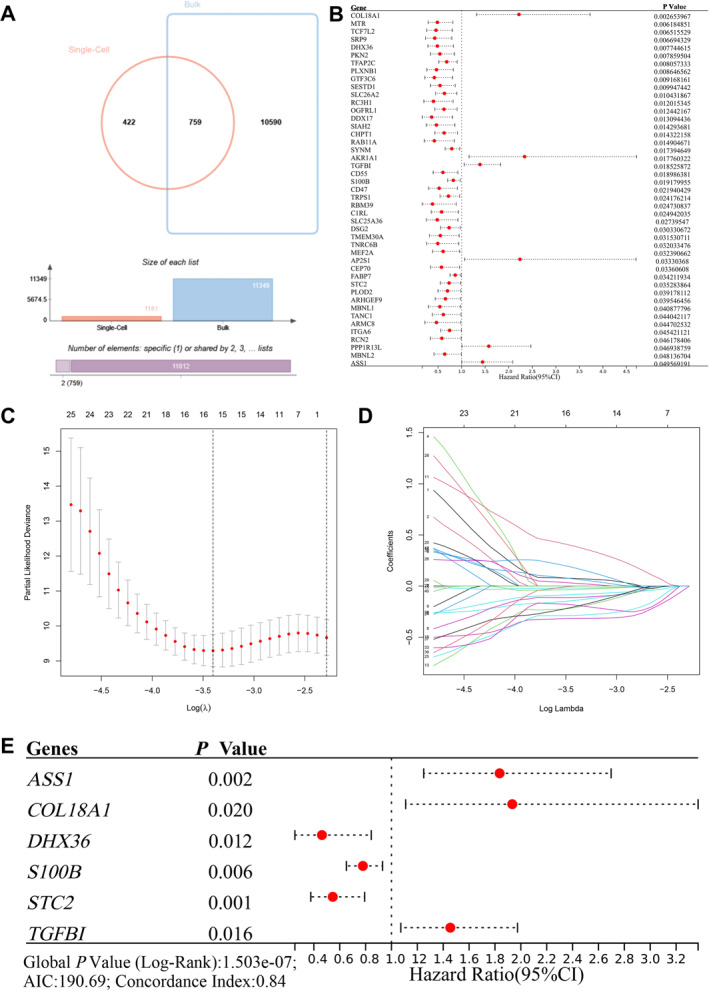
Development of an anoikis‐related gene signature. (A) Venn plot showing genes common to both the anoikis‐related module at the bulk level and the anoikis‐related markers at the single‐cell level. (B) Univariate Cox regression analysis evaluation identifying 45 anoikis‐linked genes exhibiting *p* < 0.05. (C) Lasso coefficient‐Log (*λ*) distribution for 15 anoikis‐linked genes. (D) Determination of optimal log (*λ*) parameter in Lasso analysis. (E) Stepwise multivariate Cox regression analysis was utilised to identify 6 anoikis‐linked genes for establishing a prognostic indicator in patients with TNBC. For TCGA–TNBC bulk transcriptomic analysis, *n* = 146 patients.

**FIGURE 3:**
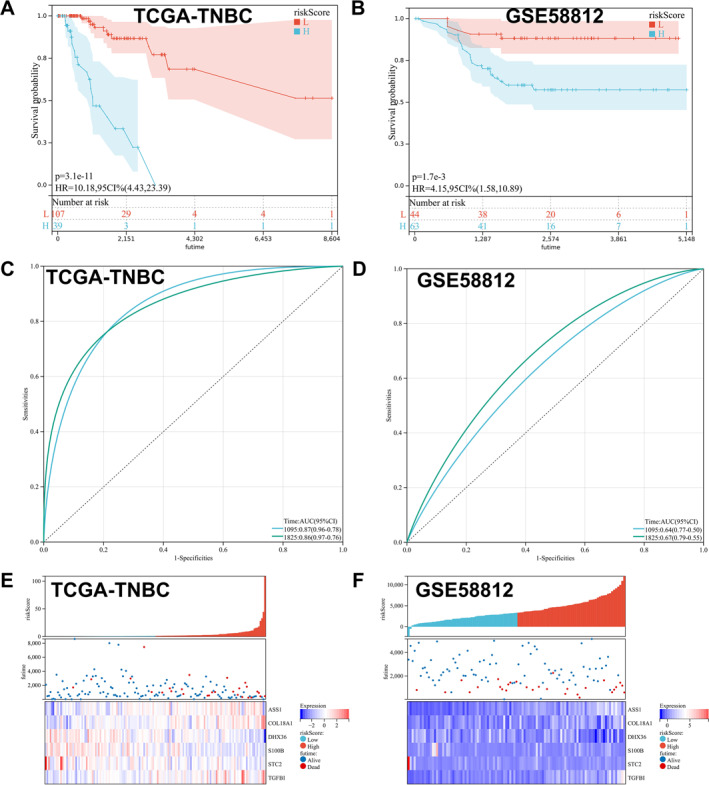
Validation of anoikis‐related gene signature. (A) Survival analysis curves depicting overall survival for patients with TNBC in the TCGA cohort. (B) Kaplan–Meier curves showing overall survival for patients with TNBC in the GSE58812 cohort. (C) ROC analysis curves demonstrating 3‐year and 5‐year OS predictions in the TCGA cohort. (D) ROC analysis curves indicating 3‐year and 5‐year OS predictions in the GSE58812 cohort. (E) Patient survival status and gene expression patterns comparing high‐risk versus low‐risk groups in the TCGA cohort. (F) Patient survival status and gene expression patterns comparing high‐risk versus low‐risk groups in the GSE58812 cohort. TCGA–TNBC cohort, *n* = 146 patients; GSE58812 cohort, *n* = 107 patients.

The analysis identified 1181 anoikis‐related markers differentiating anoikis‐resistant from anoikis‐sensitive subtypes with an FDR < 0.05 (Table S2). Within this set, 759 genes overlapped with the anoikis‐related module (Table S2) and anoikis‐related markers, establishing them as anoikis‐related genes in TNBC (Figure 2A). Univariate Cox regression identified 45 anoikis‐related genes exhibiting significant correlation with patient survival in TCGA–TNBC dataset (*p* < 0.05) (Figure 2B). Through sequential application of lasso regression (Figure 2C,D) and stepwise multivariate regression analysis, the study established a 6‐gene signature encompassing *ASS1*, *COL18A1*, *S100B*, *TGFBI*, *STC2* and *DHX36* (Figure 2E). The signature's predictive capability underwent evaluation via ROC and survival analyses utilising data from TCGA–TNBC dataset and the GEO dataset (GSE58812). Survival analysis indicated superior OS in low‐risk groups versus high‐risk groups (Figure 3A,B). The TCGA–TNBC dataset exhibited ROC curve AUC values of 0.87 and 0.86 for 3‐year and 5‐year OS, respectively (Figure 3C). The GEO dataset demonstrated AUC values of 0.64 and 0.67 for 3‐year and 5‐year OS, respectively (Figure 3D). Both datasets revealed elevated mortality rates in high‐risk versus low‐risk groups (Figure 3E,F). This evidence substantiates the exceptional prognostic value of the anoikis‐related gene signature for OS prediction in TNBC patients.

### 3.3 Investigation of the Signalling Pathways Difference Between High‐Risk Group and Low‐Risk Group

**FIGURE 4:**
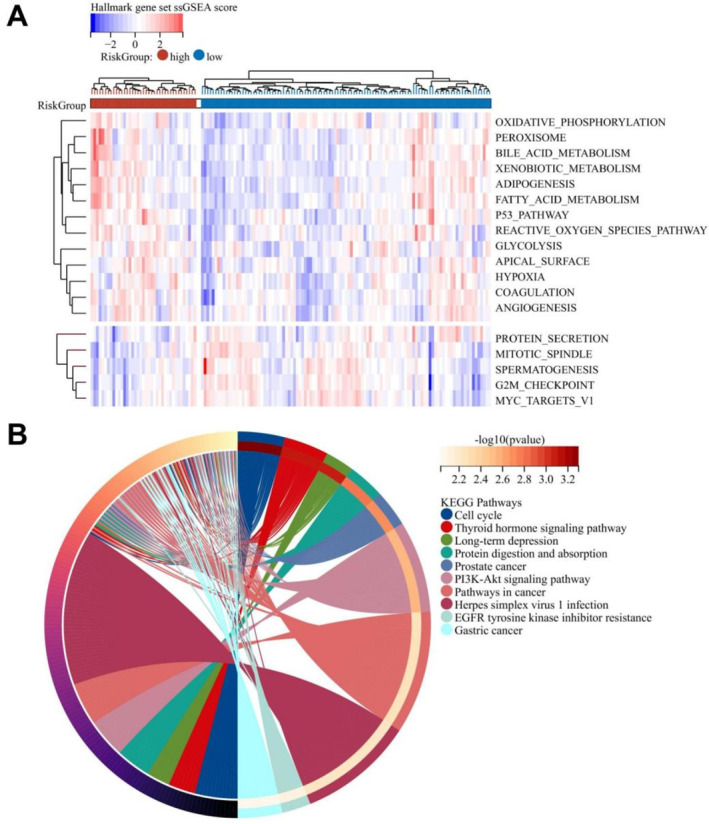
Investigation of signalling pathway differences between the high‐risk and low‐risk groups. (A) Heatmap of signalling pathways in the HALLMARK gene set significantly correlated with the anoikis risk score. (B) Circular representation of the top 10 prominent signalling pathways from the Kyoto Encyclopaedia of Genes and Genomes (KEGG) database, enriched based on differential expression analysis between high‐risk and low‐risk groups. TCGA–TNBC cohort, *n* = 146 patients.

The analysis examined associations between anoikis risk score and HALLMARK pathway ssGSEA scores, identifying significant positive correlations between anoikis riskScore and multiple resistance‐associated mechanisms, including p53 signalling, hypoxic response, glycolytic activity and angiogenesis (*p* < 0.05) (Figure 4A). Further examination through KEGG pathway analysis indicated that differentially expressed genes between high‐risk and low‐risk groups showed enrichment in tumour progression‐related pathways, particularly the PI3K‐AKT pathway (Figure 4B).

### 3.4 Evaluation of Response to Immunotherapy for Patients in High‐Risk Group and Low‐Risk Group

**FIGURE 5:**
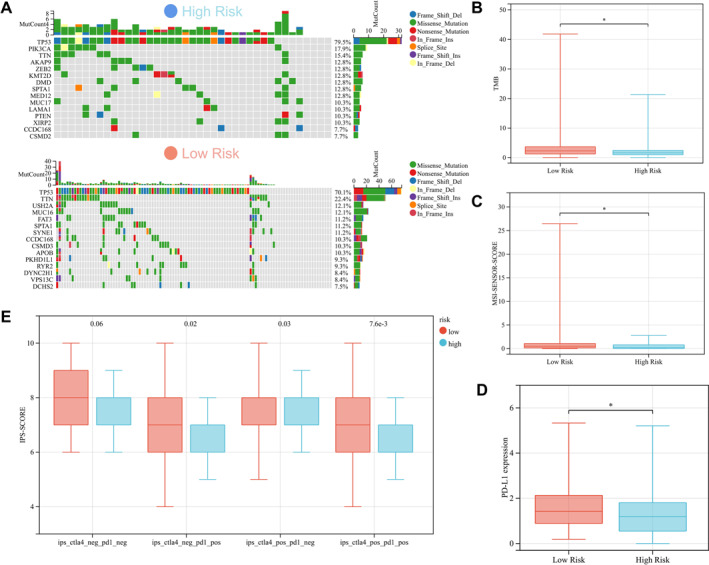
Evaluation of predicted immunotherapy response in the high‐risk and low‐risk groups. (A) Top 15 genetic alterations observed between high‐risk versus low‐risk groups. (B) Comparison of tumour mutation burden (TMB) between high‐risk versus low‐risk groups. (C) Analysis of microsatellite instability (MSI) between high‐risk versus low‐risk groups. (D) Assessment of *PD‐L1* levels between high‐risk versus low‐risk groups. (E) Comparison of immunophenoscores (IPS) between high‐risk versus low‐risk groups. *p < 0.05.

Previous research established tumour mutation burden (TMB), microsatellite instability (MSI) and PD‐L1 expression as predictive indicators of ICI response for patients undergoing immune checkpoint inhibitor (ICI) treatment [41, 42, 43, 44, 45]. Mutation frequency analysis of high‐risk and low‐risk groups in the TCGA–TNBC cohort revealed distinct patterns. The high‐risk group exhibited frequent mutations in TP53 (79.5%), PIK3CA (17.9%), TTN (15.4%), AKAP9 (12.8%) and ZEB2 (12.8%). Meanwhile, the low‐risk group displayed mutations primarily in TP53 (70.1%), TTN (22.4%), USH2A (12.1%), MUC16 (12.1%) and FAT3 (11.2%) (Figure 5A). PIK3CA and PTEN mutations activate the PI3K/AKT pathway, contributing to anoikis resistance across various malignancies. The high‐risk group demonstrated notable frequencies of PIK3CA (17.9%) and PTEN (10.3%) mutations in the TCGA‐TNBC cohort, suggesting a potential association with enhanced anoikis resistance. Additionally, analysis revealed elevated levels of TMB (Figure 5B), MSI (Figure 5C), PD‐L1 expression (Figure 5D) and IPS scores (Figure 5E) in the low‐risk group versus the high‐risk group, suggesting that patients in the low‐risk group may be more likely to benefit from ICI therapy.

### 3.5 Exploration of the TME Differences Between the High‐Risk and Low‐Risk Groups

**FIGURE 6:**
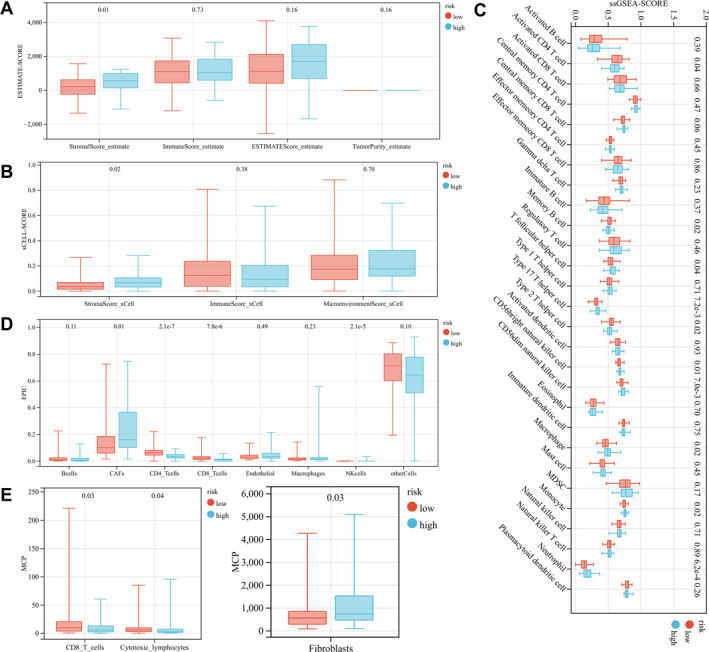
Investigation of tumour microenvironment (TME) differences between high‐risk and low‐risk groups. (A) Comparative analysis of ESTIMATE scores between high‐risk and low‐risk groups. (B) Analysis of xCELL scores between high‐risk and low‐risk groups. (C) Immune cell ssGSEA score evaluation between high‐risk and low‐risk groups. (D) Comparative examination of EPIC scores between high‐risk and low‐risk groups. (E) Assessment of MCP counter scores between high‐risk and low‐risk groups. TCGA–TNBC cohort, *n* = 146 patients.

To understand the immunotherapy‐related features in low‐risk groups, an examination of TME distinctions between groups was conducted. ESTIMATE and xCELL evaluations demonstrated elevated stromal signatures in high‐risk groups relative to low‐risk groups (Figure 6,B). Although immune signatures remained comparable between groups, cellular analyses through ssGSEA (Figure 6C), EPIC (Figure 6D) and MCPcounter (Figure 6E) indicated increased T‐cell infiltration in low‐risk groups, whereas high‐risk groups exhibited elevated CAF populations. These variations in TME composition potentially explain the more favourable immunotherapy‐related profile noted in low‐risk groups versus high‐risk groups.

### 3.6 Identification of STC2 as a Prognostic Protective Factor in TNBC

**FIGURE 7:**
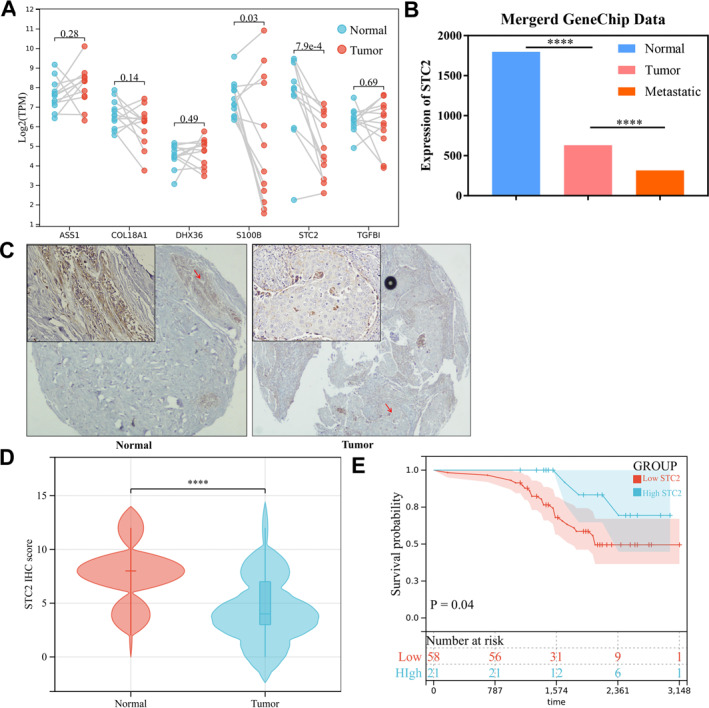
Identification of STC2 as a predictor of a protective factor for survival in TNBC. (A) Expression levels of *ASS1*, *COL18A1*, *DHX36*, *S100B*, *STC2* and *TGFBI* between TNBC samples and paired normal breast tissues. (B) Expression levels of *STC2* in normal, tumour and metastatic breast samples as analysed using merged gene chip data. (C) Representative case showing lower STC2 expression in tumour samples versus adjacent breast tissues. (D) Quantitative analysis of STC2 expression in tumour (*n* = 79) and adjacent normal breast tissues (*n* = 68). (E) Kaplan–Meier survival curves for overall survival in patients with breast cancer. ****p < 0.0001.

Among the six anoikis‐related signature genes, STC2 showed markedly significantly lower expression in TNBC specimens than in adjacent normal breast tissues (Figure 7A). Further analysis indicated that STC2 expression was lower in metastatic primary cancer tissues than in nonmetastatic cancer tissues and normal tissues (Figure 7B). To further validate STC2 expression patterns and survival implications in breast cancer, IHC analysis was performed using a breast cancer tissue microarray. STC2 expression was lower in tumour tissues than in normal breast tissues (Figure 7C,D). Moreover, patients with low STC2 expression had poorer OS in our cohort (Figure 7E).

### 3.7 Loss of STC2 Promotes Resistance in MDA‐MB‐231 Cells In Vitro

**FIGURE 8:**
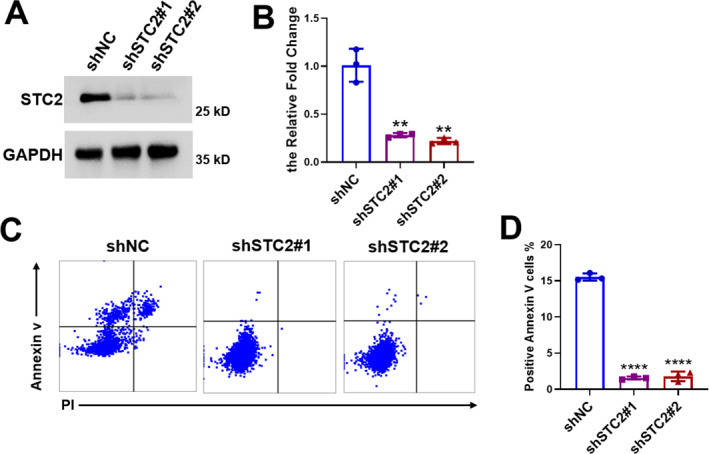
STC2 knockdown enhances anoikis resistance in an MDA‐MB‐231‐based anoikis model. (A) Protein expression analysis via western blot demonstrating STC2 suppression efficiency. (B) Gene expression analysis through RT‐qPCR indicates STC2 suppression efficiency utilising *GAPDH* as an internal control. (C) Representative flow cytometry images and (D) quantitative apoptosis rate of MDA‐MB‐231 cells with shSTC2 or shNC after 24‐h suspension culture. Data were obtained from *n* = 3.

To further investigate the role of STC2 in anoikis regulation, the MDA‐MB‐231 TNBC cell line was used as an in vitro model. The expression of STC2 was knocked down by shRNA, and expression efficacy was confirmed via western blot and RT‐qPCR analyses (Figure 8A,B). To establish an anoikis‐mimic model, ultra‐low attachment 6‐well plates to maintain cells in suspension for 24 h was used. Flow cytometry analysis evaluated the effect of STC2 on anoikis resistance. Results demonstrated that STC2 suppression led to reduced apoptosis rates in MDA‐MB‐231 cells relative to shNC controls, suggesting that STC2 reduction enhances anoikis resistance in this MDA‐MB‐231‐based TNBC model.

## 4 Discussion

Anoikis is tightly associated with the prognosis of multiple cancers, including lung cancer [46], colorectal cancer [47] and breast cancer [48, 49]. Cancer cells acquiring anoikis resistance evade death‐signalling mechanisms and persist under unfavourable circumstances [50, 51], leading to chemoresistance, distant metastasis and poor prognosis [52, 53, 54]. Numerous anoikis‐related genes have been identified as key regulators of cancer progression. For example, L1CAM facilitates epithelial‐mesenchymal transition (EMT) and promotes anoikis resistance in endometrial carcinoma, thus affecting patient prognosis [55]. IQGAP1, a novel prognostic biomarker in hepatocellular carcinoma, inhibits anoikis and promotes cell proliferation [56]. Additionally, overexpression of PDK4 has been linked to chemoresistance in lung cancer [57]. In breast cancer, several anoikis‐related factors, such as BMP4, promote anoikis resistance and chemoresistance through the BMP signalling pathway [20], whereas TDO2 enhances anoikis resistance and metastasis in TNBC via the TDO2‐AhR signalling axis [58]. Although these factors have been widely studied, establishing a multi‐gene signature derived from anoikis‐related genes remains essential for enhancing prognostic prediction in patients with TNBC beyond what single‐gene markers can achieve.

Molecular signatures derived from single‐cell gene expression data may offer greater accuracy and sensitivity compared to those identified from bulk‐level data. In this study, an anoikis‐related gene signature (*STC2*, *S100B*, *COL18A1*, *DHX36*, *TGFBI* and *ASS1*) was developed and validated using both single‐cell and bulk‐level data, exhibiting strong performance in predicting prognosis and potential chemotherapy responsiveness in patients with TNBC.

The high‐risk group exhibited notable mutations in *PIK3CA* (ranked second; 17.9%), *ZEB2* (ranked fifth; 12.8%) and *PTEN* (ranked twelfth; 10.3%) within the top 15 most frequent mutations, whereas these alterations were absent from the top 15 mutations in the low‐risk group. Analysis of differential gene expression between high‐risk and low‐risk groups indicated substantial activation of the PI3K‐AKT signalling cascade. Previous research has established that the PTEN–PI3K‐AKT pathway contributes to anoikis resistance and affects cancer outcomes and treatment response through regulation of downstream elements [59, 60, 61, 62]. ZEB2, a key mediator of EMT, disrupts adherens junctions and enables detached tumour cells to evade anoikis [63, 64, 65]. The elevated mutation frequency in anoikis resistance‐associated genes within the high‐risk group potentially accounts for the less favourable outcomes observed in these patients versus the low‐risk group.

The low‐risk group demonstrated elevated TMB, MSI and PD‐L1 expression levels versus the high‐risk group. Enhanced TMB, MSI and PD‐L1 expression correlate with superior outcomes during ICI treatment [41, 42, 43, 44, 45], suggesting that the low‐risk group may have a higher likelihood of benefiting from ICI therapy. TME differences between the high‐risk and low‐risk groups were further explored, indicating that the low‐risk group had higher T lymphoid cell infiltration and lower CAF infiltration. CAFs are known to promote tumourigenesis and immune evasion by interacting with other cell types in the TME [66, 67, 68]. CAFs facilitate anoikis resistance through metabolic support [69]. These observations suggest connections between the anoikis‐related signature and TME, potentially impacting disease progression and therapeutic response through T cell and CAF interactions. Finally, in vitro experiments confirmed that STC2 knockdown promoted anoikis resistance in MDA‐MB‐231 cells.

Despite the strong performance of our anoikis‐related gene signature in predicting TNBC prognosis and potential immunotherapy responsiveness, several limitations should be acknowledged. First, the transcriptomic data were obtained from public databases, and the predictive performance of the signature requires further validation in independent clinical cohorts. Second, due to limited resources, only the expression pattern, prognostic significance and in vitro biological function of STC2 were experimentally validated. Moreover, we did not directly assess whether the anoikis‐related signature correlates with clinical responses to anti‐PD‐1/PD‐L1 therapy because TNBC cohorts with matched transcriptomic profiles and documented ICI outcomes remain limited. Finally, the in vitro functional validation of STC2 was performed only in MDA‐MB‐231 cells. Given the substantial molecular heterogeneity of TNBC, this single‐cell‐line model may not fully represent the biological diversity of TNBC. Therefore, the role of STC2 in anoikis resistance and the predictive value of this signature for ICI response should be interpreted cautiously and further validated in larger immunotherapy‐treated cohorts, additional TNBC cell lines and in vivo models.

## 5 Conclusion

In conclusion, this investigation established an anoikis‐related gene signature with potential utility in forecasting prognosis and immunotherapy response in patients with TNBC.

## Author Contributions

**Bing Xue:** conceptualisation, data curation, writing – original draft. **Yue Bai:** conceptualisation, data curation, writing – original draft. **Wu‐Di Yang:** conceptualisation, data curation, writing – original draft. **Shi‐Zhen Li:** methodology. **Rui Qin:** methodology. **Hua Zhang:** methodology. **Hong‐Fei Wu:** funding acquisition, writing – review and editing. **Jun‐Jian Li:** formal analysis, funding acquisition, writing – review and editing. **Sen Miao:** formal analysis, funding acquisition, writing – review and editing.

## Funding

This study was funded by the Jining Key Research and Development Plan (Grant 2023YXNS057); National Natural Science Foundation of China (Grant 82272828); Natural Science Foundation of Shanghai (Grant 22ZR1450000) and State Key Laboratory of Systems Medicine for Cancer (Grant KF2129‐93), Hainan Provincial Natural Science Foundation of China (Grant 823RC563).

## Ethics Statement

This study was conducted in accordance with the Declaration of Helsinki and was approved by the Ethics Committee of the Affiliated Hospital of Jining Medical University (Approval number: 2021‐08‐C015). Written informed consent was obtained from all participants enroled in the study.

## Consent

The authors have nothing to report.

## Conflicts of Interest

This study established an anoikis‐related gene signature that may improve prognostic stratification and reflect immunotherapy‐related features in TNBC.

## Supporting information


**Table S1:** Immunohistochemistry score of STC2 and clinical information of patients with breast cancer.

**Table S2:** Identification of anoikis‐related genes in TNBC.

## Data Availability

The public datasets used to develop and validate the signature can be accessed from UCSC‐Xena (https://xenabrowser.net/datapages/) and GEO (https://www.ncbi.nlm.nih.gov/geo/). Data from the single‐cell cohort are publicly available at http://biokey.lambrechtslab.org.
