# Supplementary material for: Integrated Analysis Identifies an Anoikis‐Related Gene Signature for Predicting Prognosis in Patients With Triple‐Negative Breast Cancer
Source: IET Syst Biol. 2026 Jun 8;20(1):e70076. doi: 10.1049/syb2.70076 (PMC13245715; doi:10.1049/syb2.70076)
Supplement: Supplementary file 1 — Table S1: Immunohistochemistry score of STC2 and clinical information of patients with breast cancer. [file SYB2-20-e70076-s002.docx]

# Supplementary Table S1. Immunohistochemistry score of STC2 and clinical information of patients with breast cancer.

| Location | Patient ID | Percentage of positive tumor cells | Staining Intensity | STC2 IHC score | Age | Diagnosis | T | N | M | OS_event | OS (month) |
| --- | --- | --- | --- | --- | --- | --- | --- | --- | --- | --- | --- |
| Slide 1 | | | | | | | | | | | |
| A1 | 1509510 | 1 | 1 | 1 | 57 | T | T2 | N1 | M1 | 0 | 80 |
| A2 | 1509510 | 4 | 2 | 8 | 57 | N | T2 | N1 | M1 | 0 | 80 |
| A3 | 1533650 | 4 | 2 | 8 | 33 | T | T2 | N3 | M0 | 1 | 79 |
| A4 | 1533650 | na | na | na | 33 | N | T2 | N3 | M0 | 1 | 29 |
| A5 | 1534520 | 0 | 0 | 0 | 36 | T | T2 | N1 | M1 | 0 | 72 |
| A6 | 1534520 | 4 | 2 | 8 | 36 | N | T2 | N1 | M1 | 0 | 72 |
| A7 | 1534836 | 4 | 1 | 4 | 48 | T | T2 | N1 | M1 | 1 | 52 |
| A8 | 1534836 | 4 | 2 | 8 | 48 | N | T2 | N1 | M1 | 1 | 72 |
| A9 | 1628011 | 2 | 1 | 2 | 79 | T | T4 | N2 | M0 | 1 | 6 |
| A10 | 1628011 | 4 | 3 | 12 | 79 | N | T4 | N2 | M0 | 1 | 6 |
| B1 | 1837098 | 0 | 0 | 0 | 40 | T | T2 | N1 | M1 | 1 | 38 |
| B2 | 1837098 | 4 | 2 | 8 | 40 | N | T2 | N1 | M1 | 1 | 38 |
| B3 | 1303031 | 4 | 1 | 4 | 45 | T | T2 | N3 | M1 | 0 | 105 |
| B4 | 1303031 | 4 | 1 | 4 | 45 | N | T2 | N3 | M1 | 0 | 105 |
| B5 | 1333034 | 2 | 1 | 2 | 53 | T | T2 | N1 | M1 | 1 | 35 |
| B6 | 1333034 | 4 | 2 | 8 | 53 | N | T2 | N1 | M1 | 1 | 95 |
| B7 | 1330400 | 4 | 2 | 8 | 44 | T | T2 | N3 | M1 | 0 | 101 |
| B8 | 1330400 | 4 | 3 | 12 | 44 | N | T2 | N3 | M1 | 0 | 96 |
| B9 | 1403263 | 4 | 1 | 4 | 59 | T | T2 | N0 | M1 | 1 | 33 |
| B10 | 1403263 | 4 | 3 | 12 | 59 | N | T2 | N0 | M1 | 1 | 93 |
| C1 | 1437186 | 2 | 1 | 2 | 49 | T | T2 | N0 | M0 | 0 | 53 |
| C2 | 1437186 | 4 | 2 | 8 | 49 | N | T2 | N0 | M0 | 0 | 83 |
| C3 | 1721006 | 4 | 1 | 4 | 38 | T | T1 | N1 | M0 | 1 | 52 |
| C4 | 1721006 | 4 | 3 | 12 | 38 | N | T1 | N1 | M0 | 0 | 52 |
| C5 | 1436678 | 4 | 1 | 4 | 62 | T | T2 | N0 | M0 | 1 | 42 |
| C6 | 1436678 | 4 | 2 | 8 | 62 | N | T2 | N0 | M0 | 0 | 82 |
| C7 | 1437574 | 4 | 1 | 4 | 38 | T | T2 | N0 | M0 | 0 | 84 |
| C8 | 1437574 | 4 | 3 | 12 | 38 | N | T2 | N0 | M0 | 0 | 84 |
| C9 | 1409100 | 4 | 1 | 4 | 49 | T | T2 | N1 | M0 | 1 | 61 |
| C10 | 1409100 | 4 | 2 | 8 | 49 | N | T2 | N1 | M0 | 0 | 91 |
| D1 | 1415799 | na | na | na | 47 | T | T1 | N0 | M0 | 0 | 89 |
| D2 | 1415799 | 4 | 3 | 12 | 47 | N | T1 | N0 | M0 | 0 | 89 |
| D3 | 1408449 | 4 | 2 | 8 | 66 | T | T2 | N1 | M0 | 0 | 72 |
| D4 | 1408449 | 4 | 2 | 8 | 66 | N | T2 | N1 | M0 | 0 | 92 |
| D5 | 1424150 | 1 | 1 | 1 | 49 | T | T1 | N2 | M0 | 1 | 57 |
| D6 | 1424150 | 4 | 2 | 8 | 49 | N | T1 | N2 | M0 | 0 | 87 |
| D7 | 1436071 | 4 | 1 | 4 | 48 | T | T1 | N0 | M0 | 0 | 84 |
| D8 | 1436071 | 4 | 1 | 4 | 48 | N | T1 | N0 | M0 | 0 | 84 |
| D9 | 1740952 | 4 | 1 | 4 | 58 | T | T2 | N2 | M0 | 0 | 49 |
| D10 | 1740952 | 4 | 2 | 8 | 58 | N | T2 | N2 | M0 | 0 | 49 |
| E1 | 1219790 | 3 | 1 | 3 | 34 | T | T2 | N1 | M1 | 1 | 52 |
| E2 | 1219790 | 4 | 3 | 12 | 34 | N | T2 | N1 | M1 | 1 | 72 |
| E3 | 1605328 | 4 | 1 | 4 | 47 | T | T3 | N1 | M1 | 1 | 39 |
| E4 | 1605328 | 4 | 2 | 8 | 47 | N | T3 | N1 | M1 | 1 | 69 |
| E5 | 1640078 | 0 | 0 | 0 | 51 | T | T4 | N2 | M1 | 1 | 46 |
| E6 | 1640078 | 4 | 2 | 8 | 51 | N | T4 | N2 | M1 | 1 | 60 |
| E7 | 1607652 | 3 | 1 | 3 | 66 | T | T1 | N3 | M1 | 0 | 69 |
| E8 | 1607652 | 4 | 1 | 4 | 66 | N | T1 | N3 | M1 | 0 | 69 |
| E9 | 1202053 | 4 | 1 | 4 | 35 | T | T2 | N1 | M1 | 1 | 51 |
| E10 | 1202053 | 4 | 2 | 8 | 35 | N | T2 | N1 | M1 | 1 | 101 |
| F1 | 1518265 | 4 | 1 | 4 | 48 | T | T2 | N1 | M1 | 1 | 41 |
| F2 | 1518265 | na | na | na | 48 | N | T2 | N1 | M1 | 1 | 81 |
| F3 | 1222952 | 4 | 2 | 8 | 69 | T | T2 | N0 | M1 | 1 | 56 |
| F4 | 1222952 | 4 | 2 | 8 | 69 | N | T2 | N0 | M1 | 1 | 52 |
| F5 | 1408964 | 4 | 2 | 8 | 50 | T | T2 | N2 | M1 | 0 | 82 |
| F6 | 1408964 | 4 | 1 | 4 | 50 | N | T2 | N2 | M1 | 0 | 12 |
| F7 | 1509324 | 4 | 1 | 4 | 45 | T | T2 | N1 | M1 | 0 | 80 |
| F8 | 1509324 | 4 | 2 | 8 | 45 | N | T2 | N1 | M1 | 0 | 80 |
| F9 | 1600420 | 4 | 1 | 4 | 45 | T | T1 | N0 | M1 | 0 | 70 |
| F10 | 1600420 | na | na | na | 45 | N | T1 | N0 | M1 | 0 | 70 |
| G1 | 1509427 | 1 | 1 | 1 | 45 | T | T2 | N1 | M1 | 0 | 74 |
| G2 | 1509427 | 4 | 1 | 4 | 45 | N | T2 | N1 | M1 | 0 | 74 |
| G3 | 1513767 | 4 | 2 | 8 | 69 | T | T1 | N0 | M1 | 1 | 62 |
| G4 | 1513767 | 4 | 2 | 8 | 69 | N | T1 | N0 | M1 | 1 | 32 |
| G5 | 1109700 | 4 | 1 | 4 | 51 | T | T2 | N1 | M1 | 1 | 33 |
| G6 | 1109700 | 4 | 1 | 4 | 51 | N | T2 | N1 | M1 | 1 | 33 |
| G7 | 1733982 | 4 | 2 | 8 | 48 | T | T2 | N3 | M1 | 0 | 51 |
| G8 | 1733982 | 4 | 2 | 8 | 48 | N | T2 | N3 | M1 | 0 | 51 |
| G9 | 1434688 | na | na | na | 41 | T | T2 | N3 | M0 | 1 | 68 |
| G10 | 1434688 | 4 | 1 | 4 | 41 | N | T2 | N3 | M0 | 1 | 68 |
| H1 | 1424155 | 0 | 0 | 0 | 59 | T | T2 | N0 | M1 | 0 | 87 |
| H2 | 1424155 | 4 | 1 | 4 | 59 | N | T2 | N0 | M1 | 0 | 87 |
| H3 | 1405149 | 1 | 2 | 2 | 45 | T | T1 | N0 | M1 | 0 | 93 |
| H4 | 1405149 | 4 | 1 | 4 | 45 | N | T1 | N0 | M1 | 0 | 93 |
| H5 | 1422392 | 4 | 2 | 8 | 49 | T | T2 | N1 | M1 | 0 | 87 |
| H6 | 1422392 | 4 | 2 | 8 | 49 | N | T2 | N1 | M1 | 0 | 87 |
| H7 | 1434688 | 4 | 1 | 4 | 41 | T | T2 | N3 | M0 | 1 | 68 |
| H8 | 1434688 | 4 | 2 | 8 | 41 | N | T2 | N3 | M0 | 1 | 68 |
| H9 | 1406705 | 4 | 1 | 4 | 57 | T | T1 | N0 | M1 | 1 | 47 |
| H10 | 1406705 | na | na | na | 57 | N | T1 | N0 | M1 | 1 | 87 |
| I1 | 1437574 | na | na | na | 38 | T | T2 | N0 | M0 | 0 | 84 |
| I2 | 1437574 | 4 | 2 | 8 | 38 | N | T2 | N0 | M0 | 0 | 84 |
| I3 | 1536634 | 4 | 1 | 4 | 50 | T | T2 | N0 | M0 | 0 | 71 |
| I4 | 1536634 | 4 | 1 | 4 | 50 | N | T2 | N0 | M0 | 0 | 71 |
| I5 | 1601765 | 4 | 2 | 8 | 35 | T | T1 | N0 | M0 | 0 | 70 |
| I6 | 1601765 | 4 | 2 | 8 | 35 | N | T1 | N0 | M0 | 0 | 70 |
| I7 | 1604905 | 1 | 1 | 1 | 45 | T | T2 | N2 | M1 | 1 | 69 |
| I8 | 1604905 | 4 | 2 | 8 | 45 | N | T2 | N2 | M1 | 1 | 69 |
| Slide 2 | | | | | | | | | | | |
| A1 | 1606198 | 0 | 0 | 0 | 46 | T | T1 | N3 | M1 | 0 | 69 |
| A2 | 1606198 | na | na | na | 46 | N | T1 | N3 | M1 | 0 | 69 |
| A3 | 1619526 | 4 | 1 | 4 | 33 | T | T1 | N0 | M0 | 0 | 66 |
| A4 | 1619526 | 4 | 2 | 8 | 33 | N | T1 | N0 | M0 | 0 | 66 |
| A5 | 1626346 | 4 | 2 | 8 | 62 | T | T1 | N1 | M0 | 0 | 64 |
| A6 | 1626346 | 4 | 2 | 8 | 62 | N | T1 | N1 | M0 | 0 | 64 |
| A7 | 1627511 | 1 | 1 | 1 | 44 | T | T1 | N1 | M0 | 0 | 64 |
| A8 | 1627511 | na | na | na | 44 | N | T1 | N1 | M0 | 0 | 64 |
| A9 | 1637237 | na | na | na | 41 | T | T1 | N0 | M0 | 0 | 61 |
| A10 | 1637237 | na | na | na | 41 | N | T1 | N0 | M0 | 0 | 61 |
| B1 | 1643118 | 3 | 1 | 3 | 50 | T | T1 | N1 | M0 | 1 | 60 |
| B2 | 1643118 | 2 | 1 | 2 | 50 | N | T1 | N1 | M0 | 1 | 60 |
| B3 | 1713752 | 4 | 1 | 4 | 52 | T | T2 | N0 | M0 | 1 | 55 |
| B4 | 1713752 | 4 | 2 | 8 | 52 | N | T2 | N0 | M0 | 1 | 55 |
| B5 | 1724411 | 4 | 1 | 4 | 39 | T | T2 | N0 | M0 | 0 | 53 |
| B6 | 1724411 | 4 | 2 | 8 | 39 | N | T2 | N0 | M0 | 0 | 53 |
| B7 | 1725049 | 1 | 1 | 1 | 68 | T | T3 | N0 | M0 | 1 | 23 |
| B8 | 1725049 | 4 | 1 | 4 | 68 | N | T3 | N0 | M0 | 0 | 53 |
| B9 | 1728966 | 0 | 0 | 0 | 58 | T | T2 | N1 | M0 | 0 | 52 |
| B10 | 1728966 | na | na | na | 58 | N | T2 | N1 | M0 | 0 | 52 |
| C1 | 1743628 | 4 | 3 | 12 | 34 | T | T2 | N3 | M0 | 0 | 49 |
| C2 | 1743628 | 4 | 2 | 8 | 34 | N | T2 | N3 | M0 | 0 | 49 |
| C3 | 1747260 | 3 | 2 | 6 | 53 | T | T1 | N0 | M0 | 0 | 48 |
| C4 | 1747260 | na | na | na | 53 | N | T1 | N0 | M0 | 0 | 48 |
| C5 | 1748330 | 4 | 2 | 8 | 48 | T | T1 | N1 | M0 | 0 | 47 |
| C6 | 1748330 | na | na | na | 48 | N | T1 | N1 | M0 | 0 | 47 |
| C7 | 1748484 | 4 | 1 | 4 | 66 | T | T2 | N0 | M0 | 1 | 47 |
| C8 | 1748484 | 4 | 1 | 4 | 66 | N | T2 | N0 | M0 | 1 | 47 |
| C9 | 1748488 | 0 | 0 | 0 | 38 | T | T1 | N0 | M0 | 0 | 47 |
| C10 | 1748488 | na | na | na | 38 | N | T1 | N0 | M0 | 0 | 47 |
| D1 | 1808868 | 4 | 2 | 8 | 53 | T | T2 | N1 | M0 | 0 | 44 |
| D2 | 1808868 | 4 | 3 | 12 | 53 | N | T2 | N1 | M0 | 0 | 44 |
| D3 | 1809975 | 4 | 1 | 4 | 39 | T | T2 | N0 | M0 | 0 | 44 |
| D4 | 1809975 | 4 | 2 | 8 | 39 | N | T2 | N0 | M0 | 0 | 44 |
| D5 | 1814017 | na | na | na | 62 | T | T2 | N0 | M0 | 0 | 43 |
| D6 | 1814017 | 4 | 2 | 8 | 62 | N | T2 | N0 | M0 | 0 | 43 |
| D7 | 1814028 | 4 | 1 | 4 | 38 | T | T2 | N2 | M0 | 0 | 44 |
| D8 | 1814028 | 4 | 3 | 12 | 38 | N | T2 | N2 | M0 | 0 | 44 |
| D9 | 1814400 | na | na | na | 46 | T | T1 | N1 | M0 | 0 | 43 |
| D10 | 1814400 | 4 | 2 | 8 | 46 | N | T1 | N1 | M0 | 0 | 43 |
| E1 | 1822313 | 4 | 1 | 4 | 36 | T | T2 | N1 | M0 | 1 | 42 |
| E2 | 1822313 | na | na | na | 36 | N | T2 | N1 | M0 | 1 | 42 |
| E3 | 1822317 | 1 | 1 | 1 | 44 | T | T1 | N0 | M0 | 0 | 42 |
| E4 | 1822317 | 4 | 3 | 12 | 44 | N | T1 | N0 | M0 | 0 | 42 |
| E5 | 1841411 | 4 | 1 | 4 | 48 | T | T1 | N1 | M0 | 0 | 38 |
| E6 | 1841411 | na | na | na | 48 | N | T1 | N1 | M0 | 0 | 38 |
| E7 | 1844949 | 4 | 2 | 8 | 68 | T | T2 | N1 | M0 | 0 | 37 |
| E8 | 1844949 | 4 | 2 | 8 | 68 | N | T2 | N1 | M0 | 0 | 37 |
| E9 | 1844605 | 4 | 1 | 4 | 70 | T | T2 | N2 | M0 | 0 | 37 |
| E10 | 1844605 | 4 | 1 | 4 | 70 | N | T2 | N2 | M0 | 0 | 37 |
| F1 | 1615675 | 4 | 1 | 4 | 58 | T | T2 | N1 | M0 | 0 | 66 |
| F2 | 1615675 | 4 | 2 | 8 | 58 | N | T2 | N1 | M0 | 0 | 66 |
| F3 | 1616349 | 4 | 1 | 4 | 53 | T | T2 | N0 | M0 | 0 | 66 |
| F4 | 1616349 | 4 | 1 | 4 | 53 | N | T2 | N0 | M0 | 0 | 66 |
| F5 | 1748063 | 4 | 1 | 4 | 53 | T | T2 | N1 | M0 | 0 | 48 |
| F6 | 1748063 | 4 | 2 | 8 | 53 | N | T2 | N1 | M0 | 0 | 48 |
| F7 | 1844605 | 4 | 2 | 8 | 70 | T | T2 | N2 | M0 | 0 | 37 |
| F8 | 1844605 | na | na | na | 70 | N | T2 | N2 | M0 | 0 | 37 |
| F9 | 1609650 | 1 | 1 | 1 | 65 | T | T1 | N2 | M0 | 0 | 68 |
| F10 | 1609650 | na | na | na | 65 | N | T1 | N2 | M0 | 0 | 68 |
| G1 | 1432382 | 4 | 2 | 8 | 42 | T | T2 | N0 | M0 | 0 | 84 |
| G2 | 1432382 | 4 | 2 | 8 | 42 | N | T2 | N0 | M0 | 0 | 84 |
| G3 | 1606200 | 4 | 1 | 4 | 50 | T | T2 | N0 | M0 | 1 | 69 |
| G4 | 1606200 | 4 | 2 | 8 | 50 | N | T2 | N0 | M0 | 1 | 69 |
| G5 | 1621086 | 4 | 1 | 4 | 64 | T | T2 | N2 | M0 | 0 | 65 |
| G6 | 1621086 | 4 | 2 | 8 | 64 | N | T2 | N2 | M0 | 0 | 65 |
| G7 | 1626222 | 4 | 2 | 8 | 68 | T | T2 | N3 | M0 | 0 | 64 |
| G8 | 1626222 | na | na | na | 68 | N | T2 | N3 | M0 | 0 | 64 |
| G9 | 1749686 | 4 | 2 | 8 | 48 | T | T2 | N2 | M0 | 0 | 97 |
| G10 | 1749686 | 4 | 2 | 8 | 48 | N | T2 | N2 | M0 | 0 | 47 |
| H1 | 1750027 | 4 | 2 | 8 | 51 | T | T1 | N0 | M0 | 0 | 47 |
| H2 | 1750027 | 4 | 2 | 8 | 51 | N | T1 | N0 | M0 | 0 | 47 |
| H3 | 1801786 | na | na | na | 35 | T | T2 | N1 | M0 | 0 | 46 |
| H4 | 1801786 | 4 | 2 | 8 | 35 | N | T2 | N1 | M0 | 0 | 46 |
| H5 | 1521824 | 4 | 1 | 4 | 69 | T | T2 | N3 | M0 | 0 | 76 |
| H6 | 1521824 | 4 | 1 | 4 | 69 | N | T2 | N3 | M0 | 0 | 76 |
| H7 | 1831807 | 3 | 1 | 3 | 47 | T | T2 | N2 | M0 | 0 | 40 |
| H8 | 1831807 | na | na | na | 47 | N | T2 | N2 | M0 | 0 | 40 |
| I1 | 1717532 | 4 | 3 | 12 | 38 | T | T1 | N0 | M0 | 0 | 51 |
| I2 | 1717532 | 4 | 2 | 8 | 38 | N | T1 | N0 | M0 | 0 | 51 |
| I3 | 1406365 | 4 | 1 | 4 | 55 | T | T2 | N1 | M0 | 0 | 92 |
| I4 | 1406365 | na | na | na | 55 | N | T2 | N1 | M0 | 0 | 92 |
| I5 | 1428144 | 4 | 1 | 4 | 53 | T | T2 | N2 | M0 | 0 | 86 |
| I6 | 1428144 | 4 | 2 | 8 | 53 | N | T2 | N2 | M0 | 0 | 86 |

IHC, immunohistochemistry; Os, overall survival.
